# Barriers and facilitators for the implementation of delayed-prescription of antibiotics in family medicine: a qualitative study

**DOI:** 10.1186/s12913-024-12200-8

**Published:** 2025-01-09

**Authors:** Aline Rinaldi, Serena Petrocchi, Luca Gabutti, Anna Bullo, Peter Johannes Schulz

**Affiliations:** 1 Faculty of Communication, Culture and Society, Università Della Svizzera Italiana, Lugano, Switzerland; 2 Faculty of Biomedical Sciences, Institute of Family Medicine, Università Della Svizzera Italiana, Lugano, Switzerland; 3 Department of Communication and Media, Ewha Womans University, Seoul, South Korea

**Keywords:** Delayed prescription, Antibiotics, Doctor-patient communication, GPs, Attitudes, General population, Antibiotic resistance

## Abstract

**Background:**

Delayed prescription is a strategy used in various countries to reduce antibiotic overuse and contend the effects of antibiotic resistance; however this practice is not yet used in Switzerland. The present qualitative study was thus conducted to investigate Swiss patients’ attitudes towards the possible implementation of delayed prescription.

**Method:**

Five focus groups with the general population based on a fixed script of questions to elicit opinions on delayed prescription.

**Results:**

A total of 29 participants were involved (M = 39,76 years of age, SD = 15,91; 19 females). Participants naturally polarized into two distinct groups: one expressing attitudes against delayed prescription and the other in favor of such practice. One driver for their opposing stance was their pre-existing negative attitudes about the use of antibiotics. Other relevant themes contributing to the formation of one’s opinion on delayed prescription included the participants’ perceived convenience of this prescribing practice and their desired level of autonomy during and after a medical encounter. Another theme that emerged was the potential impact of these stances on the interpersonal relationship between doctors and their patients.

**Conclusion:**

The present study highlights the existence of several barriers and facilitators perceived by patients. Should delayed prescription be implemented in Switzerland, these results will inform policymakers about patients’ attitudes towards the practice. Doctors may also benefit from this study as it identifies the limitations to consider when discussing treatment options with patients.

**Supplementary Information:**

The online version contains supplementary material available at 10.1186/s12913-024-12200-8.

## Introduction

### Importance of the topic and theoretical background

According to the World Health Organization, antimicrobial resistance represents one of the top 10 global public health threats facing humanity [1]. It is sometimes referred to as a “silent pandemic” as it affects not only impoverished and developing nations but also continues to grow as a global issue, bringing consequences on the duration of hospital stays, treatment procedures, and healthcare costs [2]. Research aimed at countering this phenomenon primarily focuses on the patient-doctor encounter because antibiotic prescriptions in primary care account for 80–90% of all prescriptions in human medicine, making the healthcare sector one of the main drivers of antimicrobial resistance [3]. Among the main factors leading to overprescription are the uncertainty during the diagnostic process combined with doctors’ reluctance to take risks concerning their patient’s health, as well as the time constraints derived from an elevated number of patients, and the pressure exerted by patients to receive antibiotics [4–6]. Findings from the numerous studies conducted to elaborate on the reduction of antibiotic usage [7–11] reveal that behavior change interventions show the most promising results and that one of the main drivers for antibiotic overprescription is often located at the interpersonal level between patient and provider. On a similar level, Foley and colleagues [12] demonstrated that effective communication is a tool that healthcare providers can use during their encounters with patients. The quality of the communication and of the general interaction involving doctors and patients indeed affects behavior changes both in patients’ pressure for antibiotics prescription and in doctors’ over-prescription tendency.

One strategy used to mitigate the issue of antibiotic over-prescription that aligns well with these results is that of delayed prescription. This practice, which consists of issuing a prescription of antibiotics that will be available to the patient only after a waiting period [13, 14] represents a strategic compromise between acknowledging patient expectations and practising judicious antibiotic stewardship [15]. This means that delayed prescription entails good negotiating skills, which are in turn related to maintaining a trusting relationship [13–17].

Research on delayed prescription uses the Advice Response Theory (ART) [18] as a theoretical framework. The politeness theory [19], from which ART is derived, states that advice can be perceived as either a threat to the recipients’ face or an honoring act. One notion from this theory states that individuals perceive advice as effective only when they find it feasible and devoid of significant limitations. In such cases, they are more motivated to implement the advice in their actions, such as delaying the purchase of antibiotics for a few days, assuming a wait-and-see behaviour in the meantime. Following the principles of ART and politeness theory, the delayed prescription is thus a strategy highly dependent on the doctor’s ability to discuss with patients the pros and cons of this prescribing method and to acknowledge patients’ attitudes.

As demonstrated by quantitative research, delayed prescription is generally well accepted by patients, and it does not entail a significant difference in the severity of symptoms or convalescence days [10–12]. Moreover, it is typically employed for diagnosis related to respiratory tract infections, as the latter encompasses most illnesses for which antibiotics have been demonstrated to have limited effectiveness. [20].

### The present study

Although data from the Swiss Centre for Antibiotic Resistance (ANRESIS) show that Switzerland is one of the countries that report less antibiotic use in Europe [21], the Swiss healthcare system is also burdened by the problem of antibiotic resistance [22–24] and the introduction of delayed prescription might represent a practical solution. However, while in other countries various studies have already been conducted to investigate the barriers and facilitators perceived by patients [15, 25–28], given the peculiarities of the various national healthcare systems, as well as the cultural differences in the doctor-patient relationship preferences and management [29], a possible implementation in Switzerland requires an investigation also from the point of view of the Swiss patients.

In addition, in countries where the delayed prescription is already implemented, patients are accustomed to its use. Because in Switzerland the practice is not currently adopted, it is still unknown to most of the population. This means that the present study will permit to expand the knowledge on delayed prescription also from a theoretical point of view, exploring the perceptions of participants newly introduced to the theme and whose opinion is unbiased.

The main aim of the present study is, therefore, to explore the attitudes of the Swiss general population towards delayed prescriptions in family medicine and the perceived strengths and limitations of the practice. Findings from this research will increase doctors’ understanding of patient-perceived limitations, encouraging more effective discussions to identify patients’ attitudes toward antibiotic prescription and inform Swiss policymakers, showing both the best practices and the critical aspects related to the strategy of delayed prescription.

## Method

### Study design

The qualitative study design involved focus group discussions with the general adult population, with participants not professionally involved in the healthcare sector. Focus group discussions were selected as the method for data collection because they allow for interactivity between participants, and active discussions guided by the researchers, which may generate topics that the researchers were previously unaware of.

This study was conducted as part of COMETA (COmunicazione MEdica e Terapia Antibiotica), a larger project studying the communication processes determining adherence to the antibiotic regime in the context of family medicine and to be a useful support to doctor-patient relationships and informing medical decision-making.

### Procedure

From May 2023 to September 2023, participants were invited for scheduled focus group discussions at USI University (Università della Svizzera Italiana); the last focus group was held online because this modality better suited the availability of participants. Participants were selected as a starting point among acquaintances of one of the researchers involved, and later, the selection was expanded through snowball sampling and word of mouth. All participants were sent an official email with the invitation to take part in the study. A summary of inclusion and exclusion criteria is reported in Table 1.

**Table 1:** Inclusion and exclusion criteria for participants

Inclusion criteria	Exclusion criteria
Being over 18 years old Residing in Switzerland Speaking Italian	Having an educational background in the medical field

The focus group discussions were guided by two female researchers of the study (SP and AR); one is a senior researcher with extensive experience with qualitative research approaches, and the other is a PhD student. Both took turns in being either the moderator or the assistant. The role of the mediator was to prompt the discussion following the guidelines, while the assistant aimed to take field notes and help manage turns for speaking. No person other than the researchers and the participants was present in the room during the focus groups and no pilot test were performed.

Participants were welcomed, information about the focus group was provided, and they were asked to sign the consent form. Participants received only basic information about the study and were told that no prior preparation was needed to attend the focus groups; specifically, the practice of delayed prescription was not mentioned beforehand to allow for genuine and objective interactions and avoid biases. Before starting the conversation, participants answered a brief questionnaire requiring socio-demographic information and insights on their previous use of antibiotics. The researchers introduced themselves at the beginning of the discussion; they reported only information about their professional background and introduced some basic information about the reasons behind the project.

In the first part of the discussion, participants were asked to share their experiences with antibiotic therapies and to reflect more generally on the benefits and negative aspects for society tied to the use of this type of medication. The second part was dedicated to the theme of delayed prescription, which was introduced by a description read by the assistant and followed by a request for all the possible benefits and limitations of this strategy. Two different scenarios have been developed, presenting different symptoms to prompt participants to express their opinions; in many cases, the conversation flowed naturally without introducing any hypothetical situation. To conclude the discussion, participants were asked to reflect on their relationship with their doctors and the chances that receiving delayed prescriptions could affect the quality of the relationship. The overall duration of the discussion was about one hour.

The guided track for the focus groups was based on the principles of susceptibility, severity, benefits, and barriers as explained by the Health Belief Model to elicit a discussion [30]. Moreover, according to ART theory, the perceived limitations towards delayed prescription are among the elements that could deter patients from adopting the appropriate behavior for this practice, potentially leading to low adherence to antibiotic therapy. Therefore, this aspect has been included in the guide track of the focus group. To get a more comprehensive idea of the contents of focus groups, please refer to the guidelines of the focus group discussions (Supplementary material A).

Each focus group was recorded with participants’ consent and subsequently transcribed for content analysis. The participants were not contacted again, no repeated interviews were performed, and participants were not asked to review the transcripts or give feedback on the findings. To maintain confidentiality and anonymity, any information that could be linked to the identity of individual participants was removed and replaced with codes. A specific code was assigned to each participant, as a combination of a P-number, standing for participants (P1; P2; P3; …), and a FG-number, standing for focus group (FG1; FG2; FG3; …). These identifying codes are included at the beginning of each direct participant quotation.

To determine the sample size of qualitative studies, it is suggested that data collection should continue until the point of “data saturation” [31]. Previous research [31–34] indicates that conducting four to six focus groups should lead to data saturation, consequently, we estimated that five focus groups would be sufficient to reach data saturation. An inductive thematic analysis was conducted independently by two researchers. The researchers identified sections of the text and assigned them concise codes that encapsulated the content. These codes were derived from the data and were not identified in advance, they represented aspects that surfaced during the discussions and were deemed relevant by the group members, this is also the reason why the researchers did not have a pre-established coding tree to adapt to. The codes were then organized into themes and compared between the coders. In the final phase, the coders engaged in a discussion to finalize the names of the themes. No program was utilized for the analysis of the results, the researchers each resorted to Microsoft Word and Excel for the classification of themes.

This study received approval from the relevant ethical committee (2022–01965, Rif CE TI 4225).

## Results

### Participants

Twenty-nine individuals participated in a total of five focus groups. The participation rate was 96.7% as it was originally planned to have six participants for each focus group for a total of 30 participants. One participant agreed to take part in the focus group but never showed up and never gave an explanation. All participants showed basic knowledge about antibiotics and declared to have used them at least once in their lives. The analysis of the fourth and fifth focus groups revealed a redundancy in the themes. Hence, it was decided that no additional data was needed.

Sociodemographic information about the participants is reported in Table 2.

**Table 2:** Sociodemographic information about participants

Characteristics	Options	%	Range / M (SD)
Age			25 – 70 years old / 39.76 years (15.91)
Gender	Female Male Other I do not want to specify	65 35 - -	
Educational level	Middle school diploma High school diploma Bachelor Master Postgraduate qualification Professional training	- 14 28 48 3 7	
Civil status	Married / with a steady partner Single	59 41	
Self-assessed health status	Very poor Poor Average Good Very good	- 10 32 48 10	
Having children	Yes No	38 62	
Children use of antibiotics during the last year	Yes No I don’t remember	27 73 -	
Presence of a chronic condition	Yes No I do not want to specify	17 83 -	
Use of antibiotics during the last year	Yes No I do not remember	45 45 10	

**Fig. 1:**
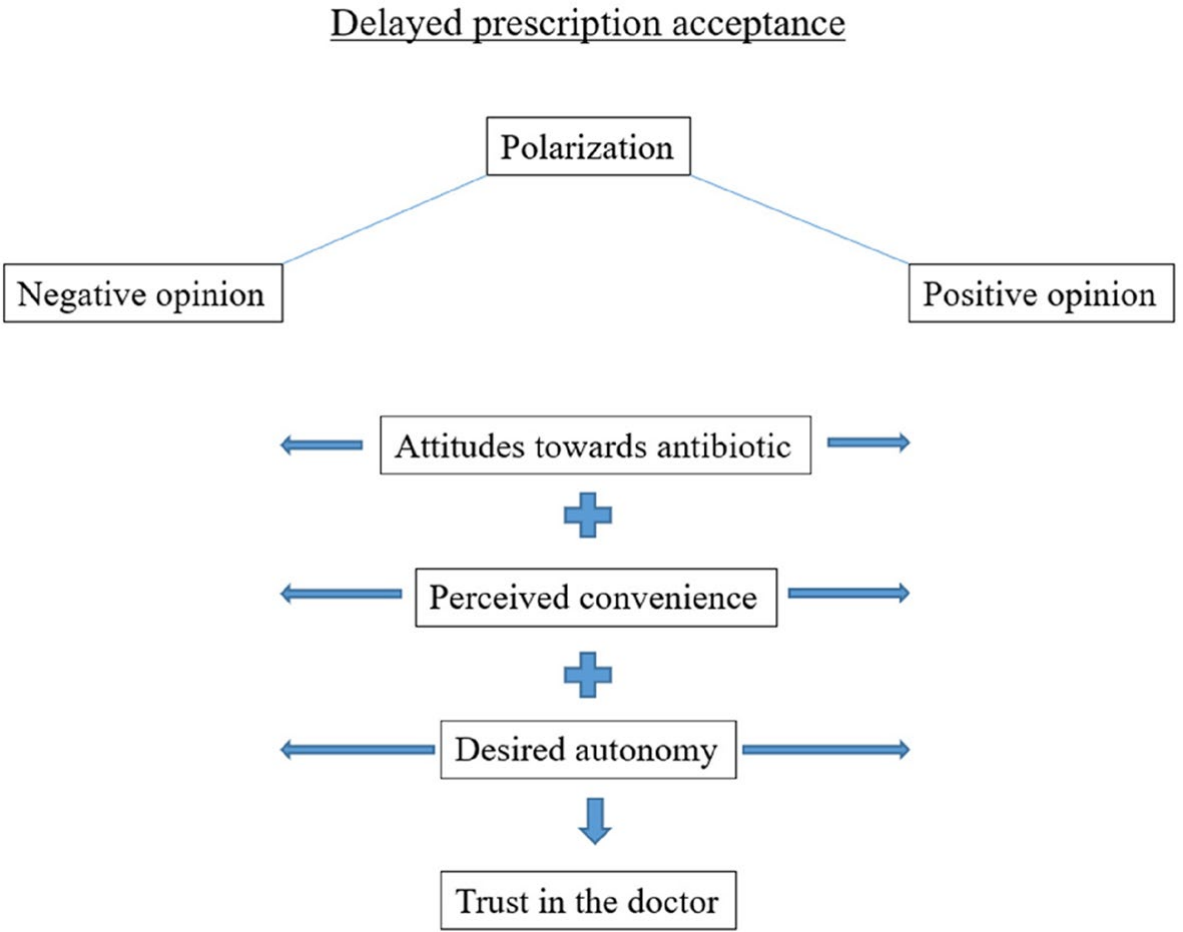
Representation of the principal themes emerged in the focus group discussion

Focus group discussions revealed that people were polarized on their attitudes towards antibiotic prescription, their perception of the convenience of delayed prescription, and their desired level of autonomy when it comes to the management of an illness. All of them have an impact on the doctor-patient relationship and the level of trust with the doctor, as presented in the scheme (Fig. 1).

#### Attitudes

A clear division among the participants was observed regarding this prescription approach, with comments generally falling into either highly positive or distinctly negative attitudes.

Some negative stances:

P1/ FG1: I don’t know when I go to the doctor, I want to have an answer. I want my answer, and I want my solution.

P2/ FG1: I don’t want to be the one deciding on this matter, I didn’t study medicine, and I shouldn’t be the one deciding in a field I am not familiar with.

P3/ FG2: […] You just delay the purchase, but they will still go and get it even if the symptoms have gotten better.

Some positive stances:

P1/ FG1: […] giving yourself a few days and waiting without having to return to the doctor to get a prescription for antibiotics is not a negative thing.

P6/ FG2: A three days-wait is perfect.

P5 / FG4: I would appreciate if I was given the chance to recover at home with milder medications than antibiotics, and if I see it is not working, then I will take them.

One factor that appears to exert a substantial influence on individuals’ initial attitudes to the concept of delayed prescription is their pre-existing expectations of receiving an antibiotic prescription. The first group of participants described positive expectations of receiving the antibiotic and experienced a sense of gratification when these expectations were met. Individuals belonging to this category typically exhibit a more adverse reaction and a negative response to delayed prescription as it impedes their immediate fulfillment of initiating antibiotic treatment as quickly as possible. Some participants in this group alluded to potential feelings of anxiety related to managing their health condition.

P2/ FG1: I mean, I would be anxious thinking that it could worsen […] there would be some anxiety […] I do not want to be the one deciding on something that I don’t know anyway.

P2/ FG2: I mean, I am the opposite and also against alternative medicine. If I need to heal, I want to heal instantly. If I have to take an antibiotic, I will take it. […] As of today, neither me, nor my husband or our children needed antibiotic a lot, but when it was necessary, I gave it to them immediately. It’s not that I looked for alternatives.

P2/ FG4: I have already tried many times to tell myself to not take the antibiotic and wait, but then it would worsen and I had to go to the emergency room, so, knowing myself I know that for that problematic I don’t want any other solution.

On the other hand, a category of individuals who adopt a more cautious approach was identified, not only concerning antibiotic usage but on pharmaceuticals as a whole. These individuals place a high value on introspection and the ability to listen to their own body’s signals. They prioritize the deliberate choice to delay medical consultation when faced with health concerns, granting their immune system the opportunity to autonomously respond to illness.

P3/ FG1: By principle, I also tend to take it only if it is necessary, in the sense that I try to let my body react first. If I see that it doesn’t work in the end and the doctor recommends it, then yes, I trust their judgment.

P6/ FG2: I have always tried to use them in a very limited manner, and only in those cases when bacteria reached too high levels, at that point I feel obligated, both towards my children and myself, to figure out how to ensure that a certain type of infection doesn’t become too…I mean, it doesn’t exceed a certain limit.

#### Convenience

This term serves as an umbrella concept that encompasses various facets. On a micro-level, participants talked about practical aspects of their daily lives that relate to delayed prescription of antibiotics, such as the necessity for regular in-person attendance at work or school or the time and energy spent to go to a medical visit. On a macro-level, participants referred to healthcare costs. On both levels, participants exhibited a polarization allowing for the depiction of two contrasting groups.

Beginning with the aspect of lifestyle, some participants have perceived the practice of delayed prescription as inherently inconvenient, particularly in cases where their occupational circumstances do not afford hybrid modalities or remote working from home. In such instances, participants have described delayed prescription as an impediment to their work life and professional or academic commitment but also as an impediment to a fast recovery.

P4/ FG1: If I have a concert in two days, I cannot wait for two days for the antibiotic.

P2/ FG4: I would not agree, for one reason, because sadly in our society you always have to work, work and be present, the consequence is that sometimes, because of clients or other reasons, you have to go to work, and instead of going to work while feeling like a dead man walking, I would rather get immediately the antibiotic.

On the other hand, there is a cohort of individuals characterized by a lifestyle that affords them greater flexibility in managing their schedules and workdays. This demographic includes those individuals who work consistently, or partially, from home as well as those fulfilling the role of homemakers. For these participants, the adoption of delayed prescription becomes a convenient and opportune approach to address episodes of illness.

P3/ FG3: I 100% agree, also because I find it more than convenient, moreover when I was in the USA I remember this approach, I was very comfortable with how they did things […] they were giving me that flexibility, that freedom, so I think it is an excellent system.

P5/ FG4: […] there is the parameter of work, I never took, I think, a day off for sick leave since I graduated from university, from that point of view I would say, for once if I get sick I would certainly get my sick-leave days and recover from home […] I would appreciate it if I could heal from home with some medicaments that are less strong than an antibiotic, then, if I see that it doesn’t work I will take it.

Another determinant factor that yielded two contrasting approaches is related to healthcare costs. Some participants frequently reported fear regarding the increasing costs due to the possible worsening of symptoms that might prompt individuals to seek medical attention before the delayed prescription becomes valid.

P5/ FG2: Yes, but something that will generate costs is also the medicaments that you would have to take from day one to day three […].

P4/ FG3: In many cases, there is an excessive use of emergency rooms […] because other people cannot evaluate the worsening or improving of their symptoms […] and so they go to the emergency room […]There is another topic that adds to the other mentioned, which makes me a bit mad, and it is the fact that these approaches are sold as measures to contain healthcare costs, when it is not the case at all, or just marginally, given the tendencies to send patient to see a specialized surgeon or neurologist even just for having a tingling on your hand.

P4/ FG5: And then if I feel so bad, I will go to the emergency room, I mean, if it is the weekend or something […] So by going to the emergency room I’m not sure how much the health insurance costs would decrease […]. (FG5).

Conversely, a subset of participants promptly recognized facets of convenience in the adoption of delayed prescription, given its exemption from a follow-up visit and a clinical reassessment in case of persistent or unimproved symptoms. Participants underscored that the medications employed to address the absence of an antibiotic are typically those stocked in household medicine cabinets. This approach achieves not only economic efficiency but also practical advantages concerning the temporal and energetic investment entailed in a medical consultation for a sick individual.

P1/ FG3: It is also true that you often go to the doctor and the first time they don’t give you the antibiotic and you must go a second time to get the prescription, as an investment of time, I mean, it is a bit…you get to optimize time with the delayed prescription.

P5/ FG5: Health costs, a reduction in healthcare costs […] because maybe you will not need the second visit, which is expensive and maybe you can avoid buying a medication that you don’t need.

There is a marginal element associated to costs that was mentioned by only one participant encompassing the production and disposal of expenditures incurred in pharmaceutical contexts. Delayed prescription may reduce patient uptake leading to a decrease in pharmaceutical waste generation and overall production rates. This reduction in the environmental footprint could be a first step for more sustainable healthcare practices with a positive impact on public healthcare costs.

P4/ FG1: Also, a reduction of environmental impact […] because the creation of one medicament has an environmental impact even just for the package they come in, the plastic…and anyways there are countries like Korea which give medicaments individually packaged, that is an insane amount of useless plastic.

#### Desired autonomy

A further theme that has generated divergence revolves around the expectations of autonomy in the management of illness. The first group displayed a more passive attitude, which entailed their preference of delegating relevant choices to doctors, as they recognize their authority in the field of medicine. This group is against delayed prescription because of the degree of autonomy it yields.

A second group of participants exhibited a more active role, preferring to manage their own health independently. This group was enthusiastic about the degree of autonomy that delayed prescription allows. Patients in this group have frequently reported they could imagine themselves experiencing a state of anxiety pertaining to the designated waiting period postulated by delayed prescription. This category of patients also includes individuals who go to the physician in search of a prompt and definitive resolution as they begin to experience a growing sense of concern due to persistent symptoms. Faced with the prospect of being issued a delayed prescription they casted doubts on the professionalism of the physician. Most notably, they demonstrate a lack of enthusiasm in even embracing the tools that would enable them to confront the situation, such as the indication from the doctor on how to evaluate if the symptoms are worsening. This is due to the belief that the patient’s role does not inherently entail assuming responsibility for their own therapy, especially during a period marked by physical and potentially psychological distress.

P2/ FG1: I do not want to be the one deciding on something that I don’t know anyway. (FG1)

P1/ FG1: Otherwise, I would do as my doctor says, you go on doctor Google and just cure yourself on your own, I mean, you are the one who spent much time studying medicine […] I would see it as something more reassuring, I mean seeing that my doctor is convinced and tells me “Take it” or “don’t take it” […] when I go to the doctor, I go to have an answer. I want my answer, and I want my solution. (FG1)

P6/ FG5: maybe the risk lays in it being not guided enough, as P5 was saying what does it even mean “if the symptoms worsen”? and how can one be sure that the patient knows how to take antibiotics. (FG5)

On the contrary, a distinct stance is represented by those participants displaying a positive response to the prospect of receiving a delayed prescription. These participants are those who have emphasized the role of communication as a pivotal element in the doctor-patient relationship, reporting efforts to negotiate compromises with their healthcare providers and delineating a visit dynamic that fosters an interactive exchange of ideas and expectations. Their appreciation of delayed prescription is rooted in the fulfillment of their expectations concerning convalescence: they acknowledged the trust that a physician demonstrates when issuing the patient with the agency to autonomously evaluate the administration of the therapeutic regimen. In this stance, the sense of empowerment arising from the delayed prescription practice is received with a dual sentiment of pride and a certain relief. This subset of participants would interpret the issuance of this prescription, in comparison to an immediate one, as an assurance. They referred to this practice as an affirmation that their health status is not profoundly compromised and could ameliorate even in the absence of antibiotic intervention.

P3/ FG1: It is a way to feel self-reliant, that is how I would interpret it.

P6/ FG2: I have always been bothered by this need to lower myself and put myself in the hands of the doctor and say: “ok fine”. Otherwise, we would have always followed other paths, maybe more…I mean, different paths, with medicines, perhaps with alternative medicine. But when you get to the antibiotic, you still try to ask if you can do without it. Well, in the end the choice is yours.

P4/ FG2: […]if my doctor doesn’t give me the antibiotic immediately, it is because she thinks that this illness, this cold, this sore throat could improve just by taking ibuprofen and I don’t need an antibiotic if she doesn’t prescribe it.

#### The effects of the delayed prescription on the quality of doctor-patient relationship

The juxtaposition of the two groups throughout the focus groups manifested itself even when discussing the consequence on the doctor-patient relationship in case they would receive a delayed prescription. The group displaying a negative attitude towards this kind of prescription described deleterious consequences, in certain instances even culminating in a loss of trust in the attending physician. This diminution of confidence prompts patients to cast doubts regarding the diagnostic proficiency of healthcare practitioners and some suggested even that a practice like delayed prescription would reduce the medical consultation to a form of economic transaction.

P5/ FG2: […] because it removes…it also removes the element of trust in the doctor, you don’t go anymore to the doctor for reassurance, you go, I mean, for a transaction.

P4/ FG3: […] In my opinion, a delayed prescription from a doctor who is not particularly attentive goes exactly in that direction, meaning a direction of liberalizing the use of antibiotics indiscriminately […]it is a system, as I see it, of self-service delayed antibiotic […] I see this delayed prescription as a shortcut, a shortcut intended to cut down on relationships that were once purely human, and are now all tariffed relationships. […] The fact that a doctor prescribes, that is, issues a prescription in the presence of vague, uncertain symptoms raises questions for me about the doctor's competence in terms of diagnosis.

Conversely, the group of participants who displayed a positive stance towards delayed prescription described also positive effects on their relationship with their doctors.

P3/ FG1: It is also a way to make me feel empowered, that is how I would interpret it on their part.

P6/ FG2: You become even more your own doctor […] making these suggestions is a step further because he cares about my health and this makes him…in short, it is praiseworthy on his part to give me the possibility not to take the antibiotic.

P3/ FG3: I love to be able to choose, especially when going to a doctor I ask questions and I would not accept to just take something or just do something, because my body is mine, and if someone tells me to do something with it, I want to know why and how […]Moreover, it even gives me the freedom after perhaps years of acquaintance, […] I think it also emphasize communication.

P5/ FG4: I think that if he explains it to me, I would trust him more […] because he is giving me an alternative […] I imagine there's always an alternative to not taking the antibiotic right away, right? […] it would make me trust him more.

## Discussion

The results of the focus groups highlight two opposing stances towards antibiotics in general and delayed prescription as a prescribing method which are rooted in three dimensions of the management of illness: the pre-existing attitudes towards healthcare and antibiotic consumption, the perceived convenience of delayed prescription and the desired autonomy in managing an illness. The two stances could be defined by the level of empowerment individuals have acquired within the dimension of their health status; depending on that derives their willingness to use a practice such as delayed prescription. For the population that demonstrates higher levels of empowerment and desired autonomy, the strategic delay in antibiotic usage enables them to refrain from immediate pharmaceutical measures, providing the autonomy to self-manage the convalescence period without necessarily compromising their professional efficacy or domestic responsibilities. On the other side, participants who did not manifest high levels of empowerment, nor the willingness to manage independently their illnesses, perceived the delay and the autonomy granted by delayed prescription as a burden and a reason for greater anxious feelings.

**Fig. 2:**
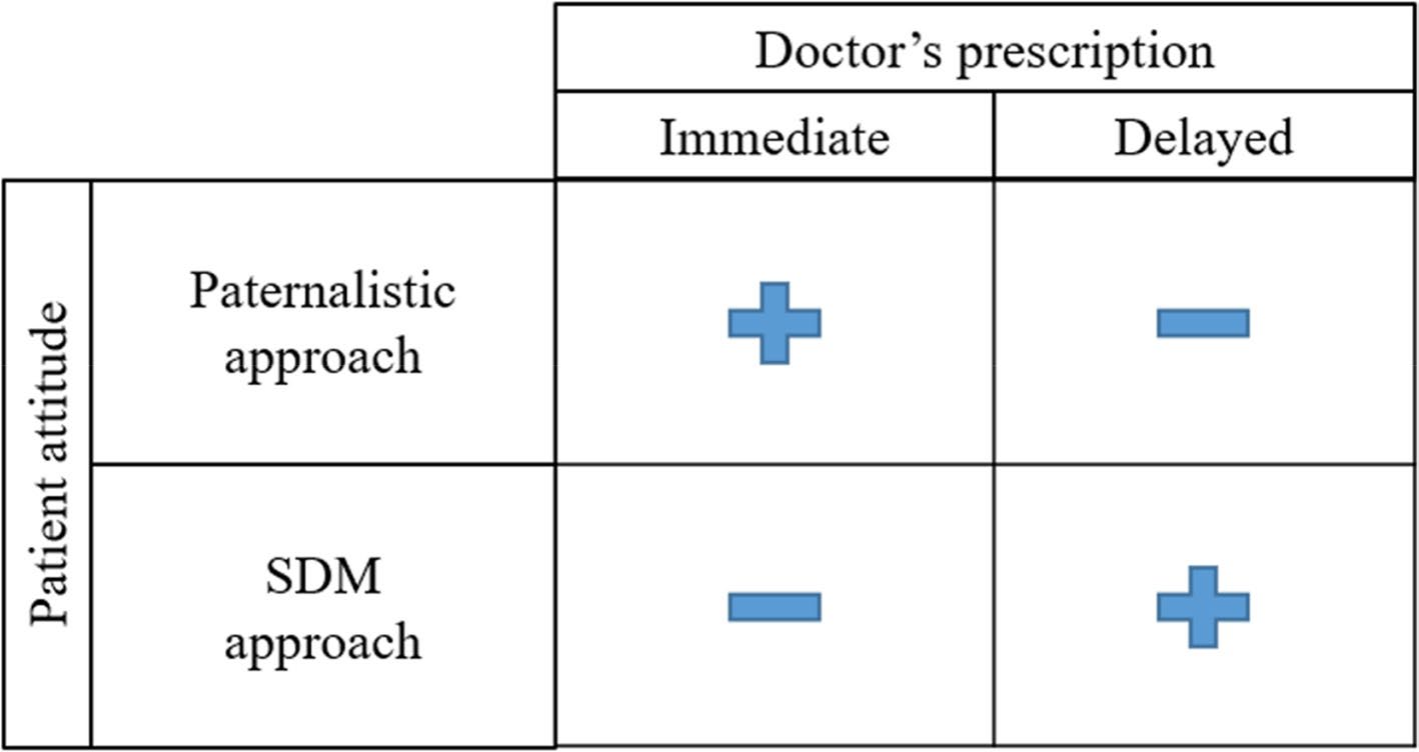
Potential interactions between healthcare providers and patients in the context of delayed prescription practices. Note: SDM = Shared Decision Making

These results underscore the persistent presence of segments within the population displaying reluctance towards gaining empowerment in this specific context. Even if the empowered patient is generally considered positively and is depicted to be the norm of today’s approach to healthcare, we should not blindly look at patients with a “one fits all” lens. On the contrary, we are once again reminded that other approaches are still represented in our society and that the healthcare system should well adapt to them to grant better results and improvements. Particularly, as our findings illustrate some of the most influential attitudes and limitations perceived by patients, this study could help doctors in efficaciously tackle the consultation. By judiciously ascertaining for whom the application of a strategy like delayed prescription might yield positive outcomes, and for whom the optimal course of action remains the issuance of an immediate prescription, doctors can avoid wasting time and resources, following the schematization proposed in Fig. 2.

Although delayed prescription is a practice that, when used consciously, can help in the management of public health expenses, our findings indicate that when patients are given this kind of prescription against their will, they might search for alternatives and second opinions. Such premature action may therefore compromise the anticipated advantages, notably the opportunity to avoid a secondary medical visit. Alternatively, individuals may resort to specialists in the field for more immediate answers or, in extreme cases, turn to emergency care facilities, which would only contribute to imposing the associated costs on public healthcare.

Going back to Brown and Levinson’s politeness theory [35] and the consequential ART theory [18], we can state that the moment a delayed prescription is issued to someone that adheres more to a paternalistic approach to medicine, it can become a threat to their face, based on Goffman depiction of the face [36], posing a challenge to the role and social value that patients attribute to themselves in the specific case of the interaction with a doctor. The contrary is true for patients who conform more to a Shared Decision-Making approach: in this case, the issuance of a delayed prescription would become an enhancement to patients’ faces as it aligns completely with the social role and worth they assign to themselves during a medical evaluation. We learn from Brown and Levinson that the only way to avoid damaging one’s face and consequently the relationship with that person is to adapt the communication style and the behavior to their expectations.

The outcomes derived from this study demonstrate that a practice largely accepted and widely used in other countries as a method for allowing patients to exert their autonomy can hide harm and larger costs when used indiscriminately. These results call for an attentive evaluation and regulation of such practice for it to be used at its best potential and avoiding repercussions at the interpersonal level of the visit, but also on the economic one, granting each patient the chance to receive the therapy that best suits their attitude. In addition, our results are in line with ART theory, proving the importance of good communication between patient and doctor.

For these reasons, the findings of this study, along with subsequent inquiries of the COMETA project, contribute to the knowledge base surrounding the dynamics of communication within the doctor-patient relationship.

## Strengths and limitations of the study

The study is part of a larger research on the dynamics between patients and providers in the realm of antibiotic prescription, which legitimates its role as a starting point: data are not gathered solely for the sake of this qualitative study but will inform and shape consequent quantitative studies in the same field. The main strengths and limitations of this first study are acknowledged below.

A first strength lies in the methodology used, which allows for an in-depth and lively discussion: the interaction between participants of focus groups generates various insights that could not be elaborated in a different setting.

The fact that we report on all the quality criteria from the COREQ quality checklist constitute a further strength of this study.

The location of the study holds both a strength and a limitation. The fact that the research takes place in a region where delayed prescription is not implemented and thus generally unknown to the general population, means that we were able to elicit unbiased genuine, and objective opinions. However, because the population is representative of the specific region in which the study takes place, results cannot be generalized to other countries. In addition, participants were requested to reflect on hypothetical grounds: while this means that collected opinions are free of bias potentially introduced by doctors or pharmaceutical companies, there is no way to know if participants would act the way they described if actually confronted with delayed prescription.

Another limitation lays in the characteristics of the participants. First, the sample was quite young, although well-balanced between gender and age. Moreover, no one displayed extreme behavior either towards requesting antibiotics or wanting to avoid them. These limitations might be tied to the method of recruitment which in its first phase occurred through acquaintances of the researchers.

Finally, another limitation of the study is the unidirectional point of view, as only individuals with no background in the healthcare sector were recruited. This gap will be filled by future research as the larger project COMETA will address the topic of delayed prescription also among family doctors.

## Conclusion

Findings from this study show that among the general population there are some shared themes that can help identify the attitudes of patients in the realm of antibiotic prescription, and more specifically around the practice of delayed prescription. These themes are the attitudes towards antibiotics, the convenience or perceived feasibility of the practice, and the desired level of autonomy. If not taken into consideration by doctors, all these aspects might have repercussions on the relationship with patients, potentially lowering the level of trust of patients.

These results can inform both general practitioners and policymakers of what are the main limitations and facilitators in accepting the practice of delayed prescription. This knowledge could help create a more effective communicative environment around the medical visit, allowing doctors to better identify the attitudes of patients and avoiding a wrongful usage of prescribing practices, which could lead to considerable waste of time and resources.

## Supplementary Information

Supplementary Material 1.

Supplementary Material 2.

## Data Availability

The dataset supporting the conclusions is available upon request to the first author.
