# Supplementary material for: Barriers and facilitators for the implementation of delayed-prescription of antibiotics in family medicine: a qualitative study
Source: BMC Health Serv Res. 2025 Jan 9;25:51. doi: 10.1186/s12913-024-12200-8 (PMC11721063; doi:10.1186/s12913-024-12200-8)
Supplement: Supplementary file 2 — Supplementary Material 2. [file 12913_2024_12200_MOESM2_ESM.docx]

**Supplementary material**

**Supplementary material A - Focus group guidelines**

As the introduction, the moderator introduced herself and the assistant, explained some basic rules of the focus group, and gave information about the aim of the research, the funding body, and the ethical approval. For space constraints, we are not reporting all this information in detail here.

Then the moderator asked the following questions:

1. *Have you ever received a prescription for an antibiotic in the past? How did you feel when you took it?*
2. *What kind of effectiveness did you experience? What kind of side effects?*
3. *Did you complete the treatment, or did you stop it earlier, and why?*
4. *What do you see as the pros and cons of taking antibiotics?*
5. *Do you know how antibiotics work? For what types of illnesses or symptoms?*
6. *Do you know what antibiotic resistance is and how it develops? How do you think it can be countered?*

The moderator introduced the delayed prescription and gave them a definition:

*So far, we have discussed the use of antibiotics, their benefits, and side effects. We talked about antibiotic resistance and reflected on completing the prescribed treatment. Now, let's talk about delayed prescriptions.* *Has anyone heard of it? Does anyone know what it means? Can we try to imagine what it is? What definition would you give?*

*A delayed prescription is a prescription issued by the doctor prescribing an antibiotic after examining the patient and detecting an infection. The delayed antibiotic prescription can only be used after a few days (usually 3), and if it is not collected after a short period, it cannot be reused in the future. Normally, the doctor leaves the prescription at the reception of their office; after three days, if the patient does not improve, they can collect the prescription and go to the pharmacy to get the antibiotic without revisiting the doctor. Delayed prescription does not replace immediate prescription, which remains a practice that doctors can adopt at any time they deem necessary. It is an alternative for specific cases or cases of uncertain diagnosis.* *In our country, this practice is not allowed. We still ask you to express your opinions.*

The moderator used the following scenarios to prompt the conversation if needed.

**Scenario 1:**

*For a few days, you have a very strong cold with a sore throat. You have difficulty swallowing, have a slight fever, general fatigue, and bone pain. You go to your doctor for a visit, and the doctor decides to prescribe an antibiotic using a delayed prescription.*

**Scenario 2:**

*For a few days, you have been experiencing abdominal pain, especially in the lower abdomen. You have difficulty urinating, with a burning sensation almost every time you go to the bathroom. You go to your family doctor who prescribes an antibiotic with delayed prescription. I ask you the same questions as before.*

**Common questions for both the scenarios:**

1. *What would you think?*
2. *How would you evaluate your doctor?*
3. *What would you think about the quality of care received? Would you be satisfied?*
4. *How would the days after the visit go? Would your symptoms improve, worsen, or remain the same?*
5. *Why do you think your doctor would prescribe an antibiotic with delayed prescription?*

The moderator espclicitally asked for benefits and negative aspects of a delayed prescription:

*Now I ask you to think about the possible benefits of delayed prescription.*

1. *What could it serve?*
2. *What benefits could it bring to the patient, the doctor, and society?*
3. *What kind of positive effects could it have? Why?*

*On the contrary, I now ask you to think about the possible negative effects.*

- *What negative effects could it have on the patient, the doctor, and society? Why?*

Conclusion:

*To conclude our discussion, I ask you to think if there are specific situations where you would never accept a delayed prescription for antibiotics and, conversely, if there are situations where you would gladly accept a delayed prescription. I refer to specific symptoms.* *What symptoms would make you refuse a delayed prescription, and what would make you accept a delayed prescription? Why?*
